# Mechanical Performance of RPC and Steel–RPC Composite Structure with Different Fiber Parameters: Experimental and Theoretical Research

**DOI:** 10.3390/polym14101933

**Published:** 2022-05-10

**Authors:** Jun Luo, Ziran Quan, Xudong Shao, Fangyuan Li, Shangwen He

**Affiliations:** 1 School of Mechanics and Safety Engineering, Zhengzhou University, Zhengzhou 450001, China; luojun@zzu.edu.cn; 2 School of Civil Engineering, Zhengzhou University, Zhengzhou 450001, China; qzr12155569@163.com; 3 School of Civil Engineering, Hunan University, Changsha 410082, China; lfy2476327981@163.com

**Keywords:** reactive powder concrete (RPC), steel–RPC composite plate, fiber type, flexural performance, crack width

## Abstract

This paper aims to explore the material properties of RPC and transverse-bending performance, as well as the crack-width-calculation theory of a densely reinforced steel–RPC composite structure with different fiber parameters. Two fiber types (straight fiber, hybrid fiber) and four fiber volume contents (2%, 2.5%, 3%, 3.5%) were selected to explore the mechanical properties of RPC materials, and the influences of fiber parameters on compressive strength, modulus of elasticity, flexural strength and axial tensile property were investigated. Eight steel–RPC composite plates with different design parameters (fiber type and reinforcement ratio) were conducted to study the transverse-bending performance of steel–RPC composite deck structures. The results show that the addition of 3.5% hybrid fibers to the RPC matrix leads to the optimum axial tensile and flexural properties. Furthermore, the failure mode, load–displacement curve, crack occurrence and propagation characteristics of the composite structure are analyzed in detail. Based on the experimental results, the calculation methods of reinforcement stress and crack width of densely reinforced steel–RPC composite structure are proposed. The calculated results of reinforcement stress and maximum crack width are in good agreement with the actual measured values, which can provide a reference for engineering design.

## 1. Introduction

**Figure 1:**
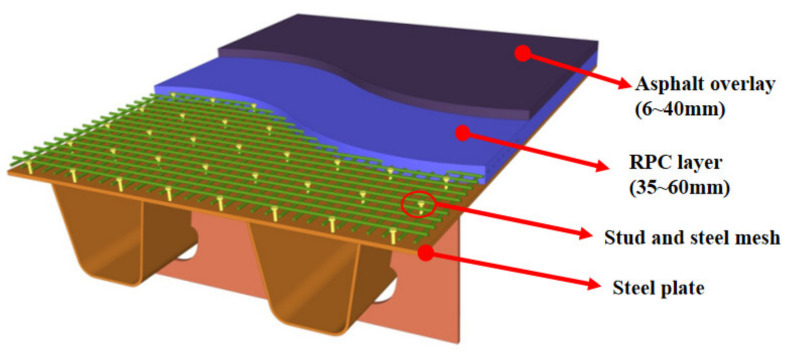
Lightweight composite bridge-deck structure with closed ribs.

Reactive powder concrete (RPC) is a kind of fiber-reinforced cement-slurry composite material composed of silica fume, cement, quartz powder, steel fiber, water reducer and other materials in a certain proportion. RPC has super high strength, toughness and excellent durability due to its internal compactness [1,2]. To solve the vulnerability of Asphalt Pavement Steel panels and fatigue cracking of steel orthotropic bridges, the project team modified RPC reinforcement and proposed a steel–RPC light composite bridge-deck structure, which connected the 35–60 mm thin RPC layer with the traditional orthotropic steel bridge deck by welding short studs [3]. The proposed lightweight deck is shown in Figure 1. Due to the outstanding mechanical properties and durability of RPC material and the combined action between steel and RPC, the novel structure significantly improved the local stiffness of the deck and reduced the stress variation in the orthotropic steel plate and pavement layer under local wheel load, which can effectively reduce the possibility of asphalt pavement damage and fatigue cracking of the steel bridge deck [4,5,6].

Usually, for clarity and design convenience, the structural systems of orthotropic steel bridge decks are divided into three basic systems: the girder system (the deck as part of the main carrying members), the deck system (the stiffened steel plate deck consisting of the longitudinal ribs, transverse floor beams and the deck plate) and the cover-plate system (the deck plate acting between longitudinal ribs) [7]. When the tire load laterally rides the U rib, the composite plate in the top of the U-rib bears a relatively large negative bending moment and the mechanical performance of the structure in this case is called transverse-bending performance, which belongs to the cover-plate system [4,7]. In this system, the steel plate and the RPC layer composite structure directly suffer the local wheel load, which means that the transverse flexural model of the steel–RPC lightweight composite deck structure can be simplified as a steel–RPC composite plate for research. Shao X.D et al. [6] showed that the design of the steel–RPC lightweight composite bridge-deck structure is controlled by the transverse cracking stress of RPC under the negative bending moment because of its lower cracking stress than that in the longitudinal direction. Furthermore, when the wheel load crosses the U-shaped stiffener along the transverse bridge direction, cracking risk of the RPC upper surface exists due to the large tensile bending stress. Therefore, it is necessary to study the transverse cracking performance of the steel–RPC lightweight composite bridge-deck structure.

In terms of material properties, Zaghloul M.M.Y [8,9] and Mohamed Y.S et al. [10] studied the fatigue, tensile and micro-hardness behaviors of fiber-reinforced thermosetting composites embedded with nanoparticles, and the experimental results showed that the addition of 4% cellulose nanocrystals to the polyester matrix leads to the optimum tensile and fatigue properties and micro-hardness results. The research conducted by Zaghloul M.M.Y [11,12,13,14] showed that different additive materials and fiber volume fractions have an important impact on the maximum flexural and tensile strengths. Wang J.Y [15] investigated the effect of strain-hardening properties on the control of crack width of RPC, and the results showed that the crack propagation of the RPC with high strain-hardening performance is slow.

Numerous scholars have carried out some experiments to study the flexural performance and the generation and development characteristics of the cracks of the RPC structure. Liu C [16] and Deng Z.C et al. [17] investigated the flexural properties of RPC beams, which showed that the RPC structure has high cracking stress with dense cracks. H.A. Rahdar [18], and Xu H.B [19] investigated the effects of the longitudinal reinforcement type and the reinforcement rate on cracking moment and crack width of the RPC members. The results showed that increasing the reinforcement rate and prestressing level can restrain the crack expansion and reduce the crack spacing. Zhang Y [20], D.Y Yoo [21] and H.M Tanarslan [22] studied the flexural performance of RPC–Normal Concrete (NC) composite beams strengthened by RPC layers, and the results showed that the RPC layers can increase the stiffness, cracking load and ultimate bearing capacity of the structure under the bending load. The increase in fiber content and reinforcement ratio can inhibit the occurrence and development of cracks. The flexural cracking performance of the RPC member is quite different from that of ordinary concrete, because the fiber type, content and reinforcement ratio have a greater impact on the flexural performance of RPC beams and RPC–NC composite beams. L Dieng [23], Shao X.D et al. [24] and Luo J [25] studied the longitudinal flexural performance of steel–RPC lightweight composite structures with closed ribs. The results showed that the composite structure had high cracking stress and when the U-rib yielded, the crack width on the RPC surface was small. Luo J [4] and Li W.G et al. [26] investigated the effects of cover thickness, reinforcement ratio, stud spacing and reinforcement arrangement on the flexural performance of steel–RPC composite slabs and described the distribution pattern of cracks. It showed that when the transverse steel bars are arranged on the upper layer and the cover thickness is small, as well as when the reinforcement ratio is high, the cracking stress of the steel–RPC composite plates is improved, and the cracks are fine and dense. Scholars have conducted certain studies on the longitudinal bending performance of RPC beams, RPC–NC composite beams, and steel–RPC composite deck structure, which showed that the reinforcement ratio, cover thickness and fiber type have a greater impact on the bending performance of RPC and the appearance and expansion of cracks. Because the transverse flexural behaviors are mainly dependent upon the local cover-plate system that includes the steel plate and the thin RPC layer, the RPC layer and steel plate are connected by welding short studs, and the RPC layer is thinner and densely reinforced; in addition, the cover thickness is smaller. These characteristics make the transverse-bending performance of steel–RPC composite deck structure quite different from these structures. However, there are few studies on the effect of the fiber parameters (fiber type and fiber volume content) on RPC material mechanical properties and transverse-bending performance of steel–RPC composite deck structures. Meanwhile, there is still a lack of theoretical research on the crack-width calculation of densely reinforced steel–RPC composite specimens, which is of great significance to the design of the lightweight composite bridge-deck structure.

This paper experimentally investigates the effects of fiber parameters on mechanical properties from the aspects of material and structure. Specifically, two fiber types (straight fiber, hybrid fiber) and four fiber volume contents (2%, 2.5%, 3%, 3.5%) were selected to explore the influence of fiber type and fiber volume content on the mechanical properties of RPC materials. At the same time, eight steel–RPC composite slabs were designed to study the influence of fiber parameters on the transverse-bending performance of steel–RPC composite deck structures. Then, the modified crack-width-calculation method is proposed based the experimental results.

## 2. Mechanical-Property Test of RPC Material

### 2.1. Materials

Two fiber types (straight fiber, hybrid fiber) and four fiber volume content (2%, 2.5%, 3%, 3.5%) were selected to explore the influence of fiber type and fiber volume content on the mechanical properties of RPC materials. The influences of fiber type and fiber volume content on compressive strength, modulus of elasticity, flexural strength and other material properties were investigated. Table 1 shows the composite parameters of steel fiber. A total of eight groups specimens were subjected to material-performance tests. According to the classification of fiber type and fiber volume content, each group of the specimens contained three four-point bending rectangular specimens (100 mm × 100 mm × 400 mm), three cubic compressive specimens (100 mm × 100 mm × 100 mm) and six modulus of elasticity rectangular specimens (100 mm × 100 mm × 300 mm).

For the axial tensile specimens, a dog-bone specimen with a 500 mm height and a minimum section length and width of 100 mm and 50 mm separately was used. Table 2 shows the specific fiber characteristic parameters. The tensile tests were divided into four groups (three specimens per group) by using fiber type and fiber combination as variables.

### 2.2. Test Plan of RPC Material

**Figure 2:**
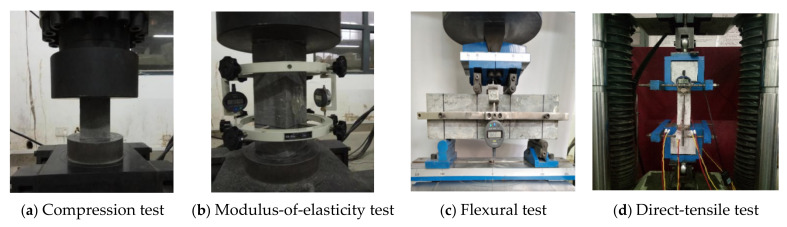
Material-performance tests.

The compressive test, modulus-of-elasticity test and flexural test of RPC were carried out according to the standard test method in the ultra-high-performance fiber-reinforced-concrete specification [27]. For the axial-tension test, the test method was based on the material test method recommended by MCS-EPFL [28]. The test photos are shown in Figure 2. In the flexural test, the dial indicator was arranged at the center of specimen to record the midspan displacement. In the direct-tensile test, strain gauges and an electronic micrometer were used to measure the strain and axial displacement of the specimen. The specimen was assembled with the fixture and fixed on a 60 t electronic servo universal testing machine for the test. The crack width, crack length, and load values were recorded during the tensile test.

### 2.3. Mechanical Properties of Material and Discussion

**Figure 3:**
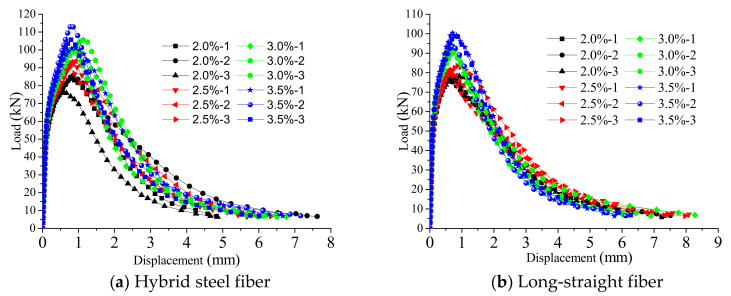
Load–midspan-displacement curves of four-point bending tests.

**Figure 4:**
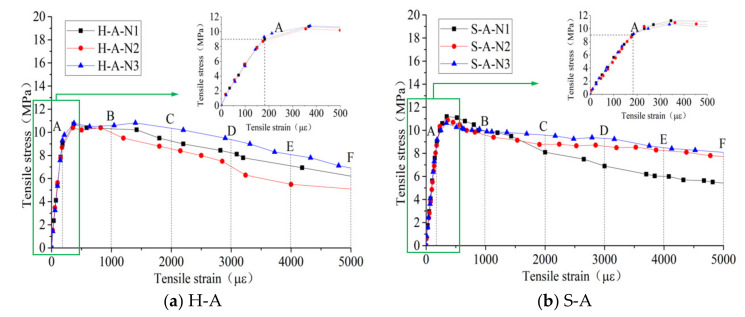

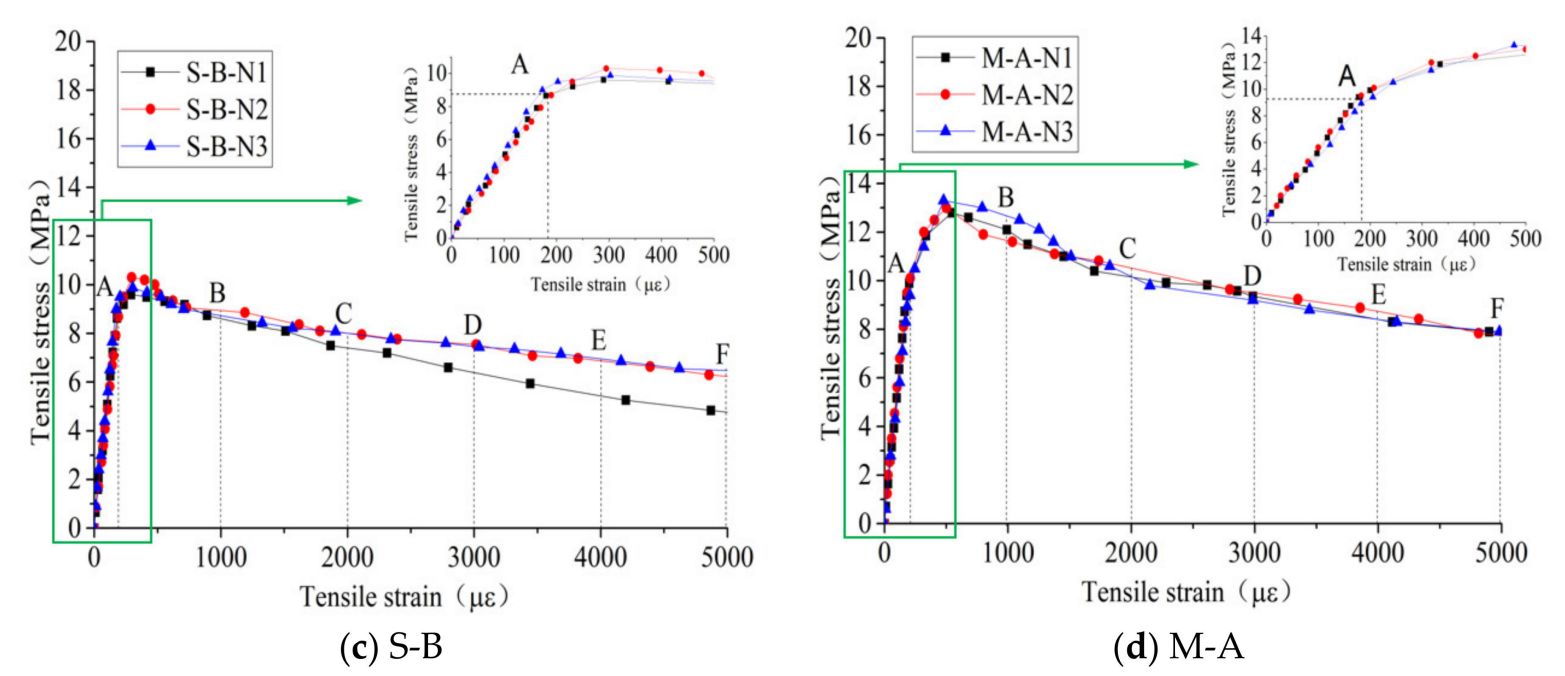
Direct-tensile-stress–strain measured curve.

**Figure 5:**
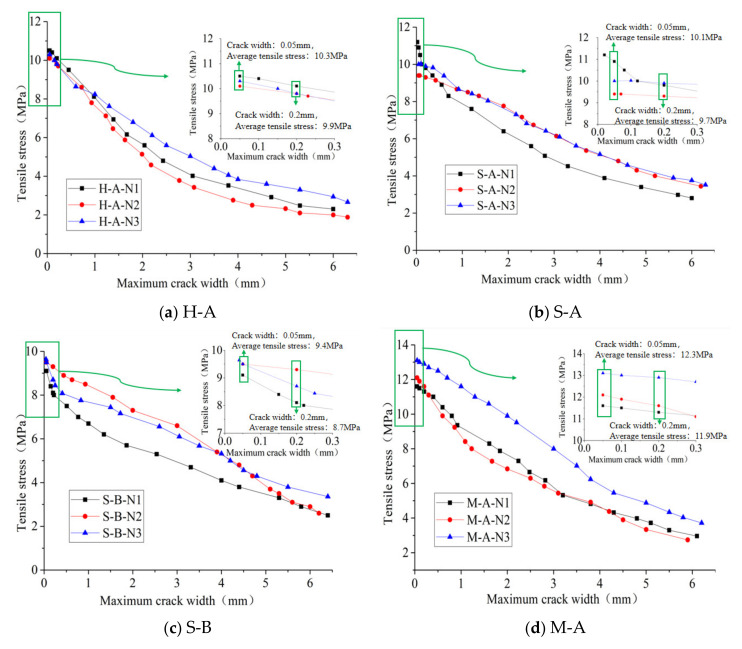
Direct-tensile-stress–crack-width measured curve.

**Figure 6:**
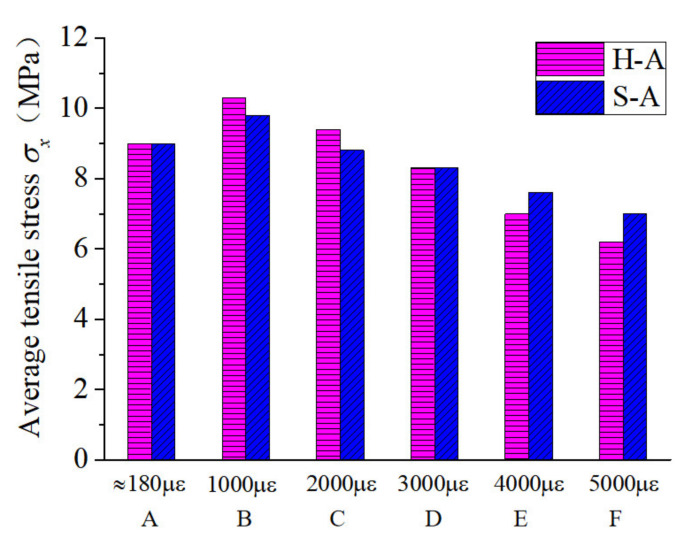
Comparison of the average tensile stress at each characteristic point for different fibers.

**Figure 7:**
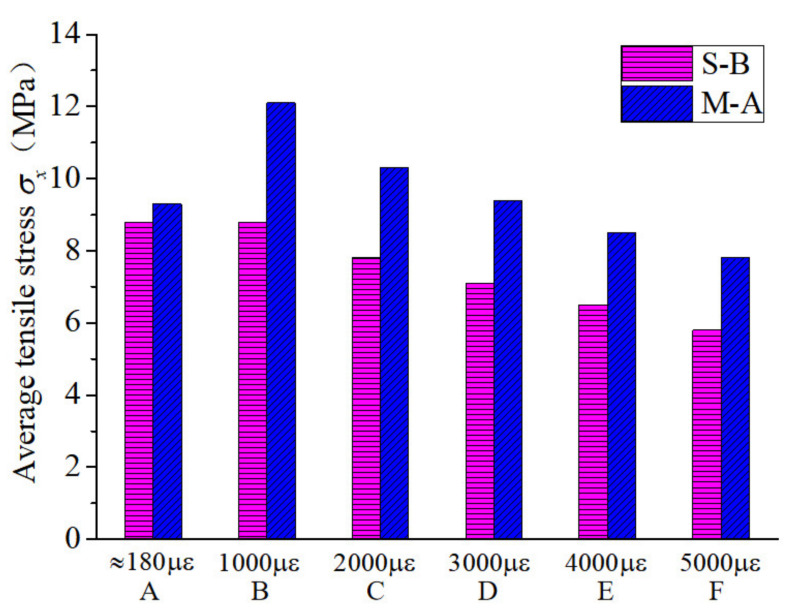
Comparison of the average tensile stress at each characteristic point with single and hybrid fibers.

The results in this section correspond to the experimental program in Section 2.1. Table 3 shows the measured results including compressive strength, modulus of elasticity and the main flexural-test results of each group of specimens. Figure 3 shows the load–midspan-displacement curves of the flexural tests. Figure 4, Figure 5, Figure 6 and Figure 7 show the results of the direct-tensile tests, which include the stress, strain and crack width.

It can be seen from Table 3 that for the long-straight fiber specimen, when the fiber volume content gradually increased from 2% to 3.5%, the compressive strength increased by 2.6%, 3.1%, and 6.2%, respectively. For the hybrid fiber specimen, when the fiber content increased from 2% to 3.5%, the compressive strength increased by 3.2%, 6.1%, and 10.8%, respectively. It shows that increasing the fiber volume content can improve the compressive strength of RPC materials, and that the hybrid fiber had a better effect on improving the compressive strength than the long-straight fiber. For the modulus of elasticity, whether it was the long-straight-fiber member or the hybrid-fiber member, the specimen with a fiber volume content of 2% had a significantly lower modulus of elasticity than the other members. When the fiber volume content was greater than 2.5%, the increase in the fiber in content had little effect on the modulus of elasticity.

Figure 3 reveals that the load–displacement curve of the four-point bending test includes three stages: the elastic stage, the crack-propagation stage and the decreasing-bearing-capacity stage. This deviation point between the elastic stage and the crack-propagation stage is called the initial bending point. With the increase in fiber content, the crack-propagation stage becomes longer during the crack-extension stage, and the ultimate load gradually increases. In the decreasing stage of the bearing capacity, the cracks gradually widen, some of the steel fibers are pulled out of the RPC matrix, thus the load rapidly decreases, and the displacement rapidly increases until the bearing capacity is lost.

The main flexural-test results in Table 3 show that for the specimens of the same fiber type, the load at the initial bending point and the load at the bending limit point increased with the increase in the fiber volume content. For long and straight fiber specimens, when the fiber volume content increased from 2% to 3.5%, the initial bending-point load and the bending-limit-point load increased by 5.5% and 26.9%, respectively. For the hybrid-fiber specimens, when the fiber volume content increased from 2% to 3.5%, the initial bending-point load and the bending limit point load increased by 19.0% and 24.7%, respectively. It can be seen that increasing the content of steel fiber can effectively increase the initial bending load and ultimate load of the member, and the effect of the hybrid fiber was better than that of the long straight fiber.

The results of direct-tensile tests are shown in Figure 4, Figure 5, Figure 6 and Figure 7. Figure 4 shows the stress–strain curves of the direct-tensile tests of RPC. The tensile-strain range in the figure was chosen from 0 to 5000 με to facilitate the observation of the direct-tensile-stress–strain curves for the different damage types. The literature [29] suggests that the mechanical condition for the strain hardening of RPC materials is $\sigma_{pc}\geq \sigma_{ca}$, where $\sigma_{pc}$ is the peak-point (Point G) stress and $\sigma_{ca}$ is the respective initial-crack-point (Point A) stress. As can be seen in Figure 4, $\sigma_{pc}$ is greater than $\sigma_{ca}$ for all direct-tensile specimens. Therefore, strain hardening can occur in RPC materials when the steel-fiber volume content is 2%, and the direct-tensile process consists of three phases: elastic phase, strain-hardening phase and stress-softening phase. Figure 5 reveals that when the fiber volume content was 2%, the direct-tensile stresses of specimens changed with the change in fiber characteristics at the same crack width, which is shown as follows: when the crack width was the same, the direct-tensile stress of end-hook fiber specimens was slightly higher than that of straight fiber specimens by 0.2 MPa, and the hybrid-fiber specimens could maintain a higher direct-tensile stress compared with the single-fiber specimens.

Furthermore, the average tensile stresses at each characteristic point with different fiber characteristics were compared and analyzed. It can be seen from Figure 6 that the fiber type had a significant effect on the reduction rate of the direct-tensile stress after the initial cracking of the RPC specimen. The initial cracking stresses of the end-hook and straight fiber RPC materials were approximately equivalent. Because of the more pronounced strain-hardening platform of end-hook fiber specimens, they had higher direct-tensile stresses than the straight fiber specimens at a strain of 1000 με (point B). When the direct-tensile strain reached 3000 με (point D), the tensile stresses were again approximately the same, after which the end-hook specimens remained below the tensile stresses of the straight specimens. During the direct-tension test of the RPC specimens, when the width of the cracks reached a certain level, the end-hook fibers were gradually straightened while causing some damage to the substrate, so the direct tension stress of the end-hook specimens decreased at a higher rate than that of the straight specimens. As can be seen from Figure 7, for samples with straight fibers, the effect of fiber type on the initial crack stress $\sigma_{ca}$ was small, but hybrid fibers significantly increased the direct-tensile stress at each characteristic point, which may be related to the fact that the hybrid fiber contained short fibers, leading to an increase in the number of fibers per unit volume and thus an increase in the bridging capacity of the fibers to the substrate.

The results of the material-performance tests show that increasing the fiber content can effectively improve the compressive strength, bending initial crack strength, and flexural strength of the specimens. Meanwhile, the hybrid fiber was more effective than the long straight type of fiber. Therefore, on the level of material performance, hybrid steel fiber with higher steel-fiber volume content is preferred in engineering applications.

## 3. The Bending Test of Steel–RPC Specimen

### 3.1. Parameter Design of Specimen

Table 4 shows the design parameters of the bending test. Eight steel–RPC composite plate bending tests were set up to study the transverse-bending performance of the steel–RPC light composite deck structure, and the test variables are the type of mixed fibers and the number of longitudinal reinforcement bars. The total volume content of mixed fibers is 3.5%.

### 3.2. Structure preparation

**Figure 8:**
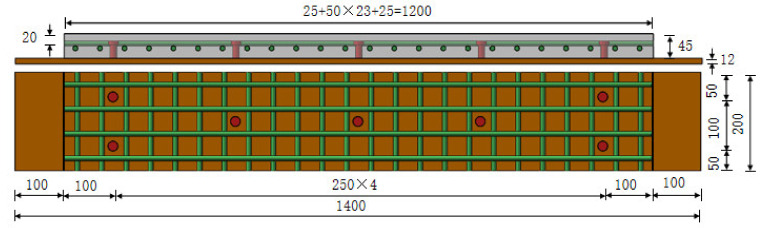
Vertical section and horizontal cross-section of specimen Z/DS45-4 (Dimensions in mm).

**Figure 9:**
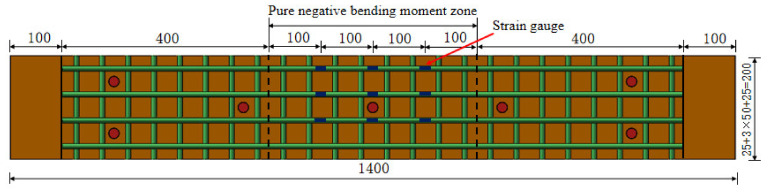
Arrangement of strain gauges on steel bars (Dimensions in mm).

The schematic diagram of the investigated structure (take Z/DS45-4 as an example) is shown in Figure 8. The structure size was 1400 mm (length) × 200 mm(width) × 57 mm (height). The thickness of RPC layer was 45 mm, the thickness of steel plate was 12 mm, and the grade of steel was Q345qC. The stud diameter was 13 mm and the height after welding was 35 mm. The steel reinforcement adopted HRB400 steel bars with a diameter of 10 mm. The longitudinal steel bars were arranged on the upper layer and the cover thickness was 15 mm. The spacing between the bars in longitudinal and transverse directions was the same. To ensure the accuracy of the test results, two identical members were set for the same variable. Nine strain gauges were mounted on the steel bars along the longitudinal direction at the four equal points of the pure-negative-bending-moment zone, as shown in Figure 9. After pouring, the members were moistened and naturally maintained for 2 days, then steam was maintained for 48 h at a temperature of 90–100 °C.

### 3.3. Loading Procedure and Instruments

**Figure 10:**
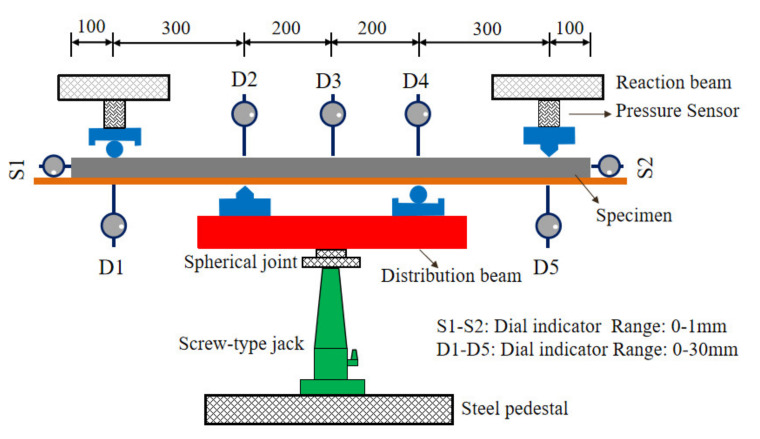
Loading diagram of the steel–RPC composite plate.

Figure 10 shows the loading diagram of the test. All members adopted the four-point negative-bending loading scheme, and the force was loaded from below by a screw jack in order to facilitate the observation of cracks on the RPC surface. The net span length of the beam was 1000 mm, and the length of the pure-bending-moment zone was 400 mm. The force was measured by the pressure sensors. The pressure sensor was arranged on each side of the member to ensure the accuracy of the results. The TDS-602 static data-acquisition instrument was used to collect the measured data of strain gauges, and the dial indicators were used to measure the deflections at the center of the span and the support with an accuracy of 0.01 mm. The slip at the contact surface between the end steel plate and the RPC was measured by dial indicators (S1 and S2) with an accuracy of 0.001 mm. The intelligent crack-width observer PTS-E40 was used to measure the crack width with an accuracy of 0.01 mm.

**Figure 11:**
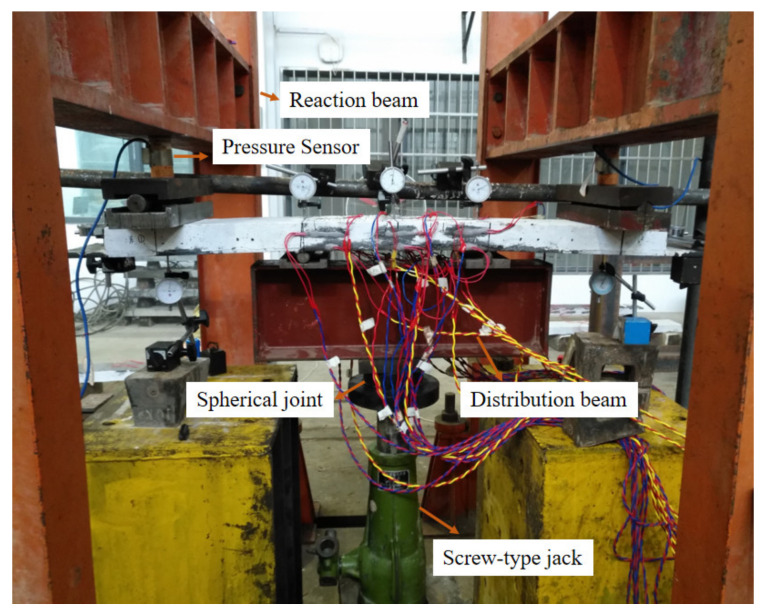
Test Diagram of Steel–RPC Composite Plate.

In the elastic phase, the loading method was force-controlled loading and the load increased by 2 kN per minute. The loading mode was switched to displacement-controlled loading when the load exceeded the linear elastic limit load, and the loading amplitude was the displacement value generated under the action of each level of load in the elastic stage. The data should be measured after the load is stable. The test photo is shown in Figure 11.

### 3.4. Test Results and Discussion of Composite Specimen

#### 3.4.1. The Main Mechanical Characteristics from Bending Test

Table 5 shows the main test results. When the crack width of the RPC layer was less than 0.05 mm, the crack was difficult to detect with the naked eye and did not affect the durability performance of the RPC member [30,31]. Therefore, the load at the top surface of the RPC with a crack width of 0.05 mm was usually defined as the cracking load in the actual structure. The nominal cracking stresses of the members were calculated according to the converted section method for the combined sections.

A comparison from Table 5 shows that for straight mixed-fiber members, when the reinforcement ratio increased from 3.5% to 5.2%, the cracking stress of the members increased by 12.4%, and the ultimate load increased by 29.2%. For the EHHF members, the cracking stress of the member increased by 33.3%, and the ultimate load increased by 25.5%. When the reinforcement ratio was the same, compared with the linear hybrid-fiber members, the cracking stresses of EHHF members with four steel bars and six steel bars were increased by 6.7% and 26.5%, respectively, while the ultimate load was almost the same. Therefore, increasing the reinforcement ratio can effectively improve the cracking stress and ultimate load of the member. Compared with the straight mixed fibers, the EHHF can increase the cracking stress to a greater extent for larger reinforcement ratios, but the effect on the ultimate bearing capacity was limited.

#### 3.4.2. Load–Displacement Curve

**Figure 12:**
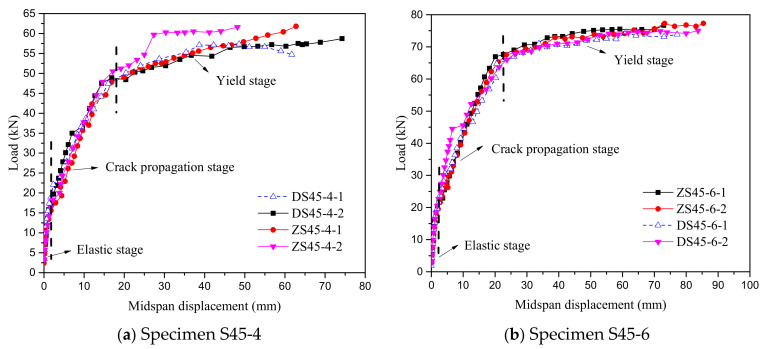
Load–midspan-displacement curves of steel–RPC composite plates.

**Figure 13:**
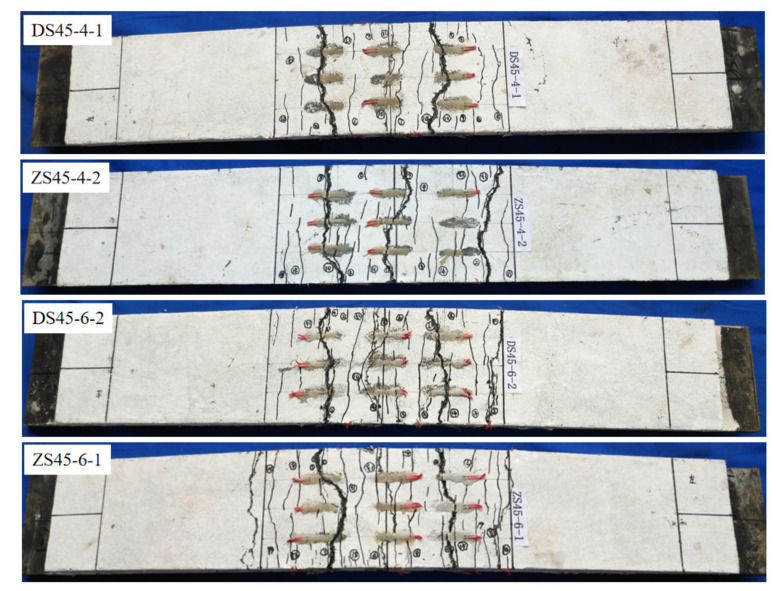
Photos of some damaged components.

The load–midspan-displacement curve is shown in Figure 12, which shows that the law of the load–midspan-displacement curve of the EHHF member is similar to that of the straight hybrid-fiber members, and both went through the elastic stage, the crack-propagation stage and the yield stage. The load–midspan-displacement curves of the two hybrid-fiber members largely overlap in the elastic phase. During the crack-extension stage, short transverse visible cracks appeared first on the top surface of the RPC layer in the purely bending section. As the load increased, the number of cracks in the purely bending section increased, the crack spacing decreased, the crack width increased, and the cracks grew laterally and developed vertically towards the bottom of the beam. As a result, the stiffness of the member decreased, and the rate of displacement increased with the load as cracks appeared on the RPC surface. The displacement of straight hybrid-fiber members was greater than that of the EHHF members under the same load. With the increase in load, the member entered the yield stage with the yield of longitudinal reinforcement. In the yield stage, the displacement increased rapidly, but the bearing capacity of the member remained basically unchanged, which indicates that the steel-reinforced RPC composite plate had good ductility and the ultimate bearing capacity was basically the same. In addition, when the longitudinal bars were close to yielding, the crack width increased rapidly. The cracks of RPC were mainly distributed within 400 mm of the pure-bending section, as shown in Figure 13.

#### 3.4.3. Analysis of the Width and Spacing of Cracks

**Figure 14:**
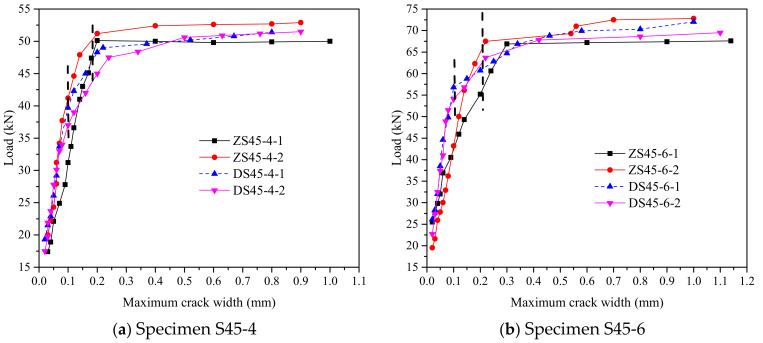
Load–maximum-crack-width curves.

**Figure 15:**
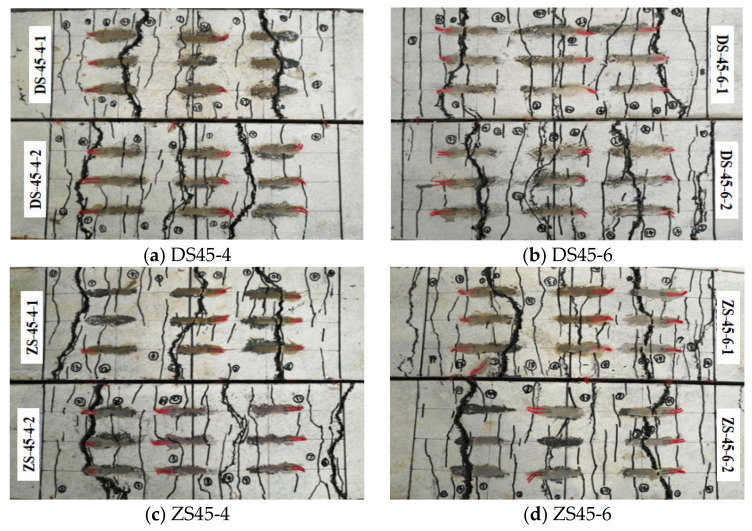
Distribution of cracks in pure-negative-bending-moment zone.

During the test, the width, length and distribution position of each crack under each level of load were recorded. Figure 14 shows the curve of load and maximum crack width on RPC surface. Table 6 shows the average crack spacing in the ultimate bearing capacity state and the number of main cracks in the final state. It can be seen from Figure 14 that when the maximum crack width was less than 0.2 mm, the crack expanded slowly. When the maximum crack width of RPC exceeded 0.2 mm, the bearing capacity of the member basically no longer increased, but the crack width expanded rapidly. For straight hybrid-fiber members, when the maximum crack width was less than 0.2 mm, the bending-load–maximum-crack-width curve approximated a straight line. While for the EHHF, when the maximum crack width was less than 0.1 mm, the load–maximum-crack-width curve approximated a straight line, and the crack-expansion speed was significantly lower than that of straight hybrid-fiber members. The main reason is that the bonding and anchoring effect between end hook hybrid fiber and RPC matrix increased because of the existence of the end hook, which can effectively prevent the initial expansion of the crack. As a result, the crack-expansion speed was significantly lower than that of straight hybrid-fiber members. When the crack width was between 0.1–0.2 mm, the speed of the crack propagation increased. After the formation of the main crack, for the EHHF members, the width of the cracks except the main crack hardly increased any more, while for straight hybrid-fiber members, the width of the cracks other than the main crack continued to increase, eventually forming 2–3 main cracks, as shown in Figure 15.

From the data in Table 6 and Figure 14, it can be seen that the cracks in the pure-bending section of the reinforced steel–RPC composite plate are densely distributed and relatively uniform in the ultimate-bearing-capacity state. For members with the same fiber type, when the reinforcement ratio increased, the cracks became denser and the average crack spacing decreased. For members with the same reinforcement ratio, the average crack spacing of straight mixed-fiber members was smaller and the cracks were denser than those of the end-hooked hybrid-fiber members.

## 4. Crack-Width-Calculation Method for Steel–RPC Composite Structures

### 4.1. Applicability Verification of Existing Codes for Crack-Width Calculation

This paper selected the Chinese technical specifications for fiber-reinforced-concrete structures based on the comprehensive analysis method (CECS38-2004) [32] and the French ultra-high-performance fiber-reinforced-concrete design code (UHPFRC-2016 code) [27] to evaluate the applicability of the crack-width calculation of the steel–RPC composite plate. Section 3.4 shows that the cracks in the steel–RPC composite plate are dense, and its distribution and expansion characteristics are different from ordinary concrete and fiber concrete. When the crack width was less than 0.2 mm, the crack width expanded slowly and the load–crack-width curve was approximately straight. When the crack width exceeded 0.2 mm, the crack expanded rapidly. Therefore, when discussing the calculation method of the crack width of the steel–RPC composite structure, the crack width was simulated to 0.2 mm.

The formulas in CECS38-2004 for calculating the maximum crack width of fiber-reinforced concrete are:

(1) $$w_{f\max}=w_{\max}\left(1-\beta_{cw}\lambda_{f}\right)$$

(2) $$w_{\max}=\alpha_{cr}\psi \frac{\sigma_{s}}{E_{s}}l_{cr}$$

(3) $$l_{cr}=1.9c_{s}+0.08d_{eq}/\rho_{te}$$

(4) $$\psi =1.1-0.65f_{tk}/\left(\rho_{te}\sigma_{s}\right)$$

where $w_{\max}$ is the maximum crack width of reinforced-concrete members; $\beta_{cw}$ is the crack-width influence factor; $\lambda_{f}$ is the characteristic value of the steel-fiber content and is equal to the fiber-length-to-diameter ratio multiplied by the volume rate; $\alpha_{cr}$ is the force characteristic factor of the member; ψ is the strain unevenness factor of longitudinal tensile reinforcement between cracks; $\sigma_{s}$ is the stress in longitudinal reinforcement; $E_{s}$ is the modulus of elasticity of reinforcement; $l_{cr}$ is the average crack spacing; $c_{s}$ is the distance from the outer edge of the outermost longitudinal tensile reinforcement to the bottom edge of the tensile zone; $\rho_{te}$ is the reinforcement ratio of longitudinal tensile reinforcement calculated on the basis of the effective tensile concrete cross-sectional area; $d_{eq}$ is the equivalent diameter of longitudinal reinforcement in the tensile zone; $f_{tk}$ is the standard value of the axial tensile strength of concrete.

**Figure 16:**
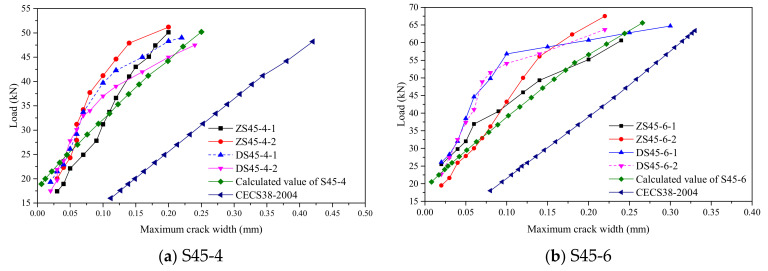
Load–Maximum-Crack-Width Curve.

Figure 16 shows the comparison between the calculated crack-width values according to CECS38-2004 and the measured crack-width values. It can be seen that the relative error between the measured value and the calculated value under the same load is relatively large and the calculated crack width of the steel–RPC composite plate based on CECS38-2004 is too conservative. The main reason is that the RPC material is dense, the bond strength between the steel fibers and the RPC matrix is greatly improved, and the bridging effect of the steel fibers shares part of the tensile force and reduces the stress on the reinforcement at the crack. RPC does not cease to function after cracking, and it can reduce the required transmission length and shorten the crack spacing. As can be seen from the above, the steel–RPC composite plate has denser cracks and the key parameters such as stress of the reinforcement bar and average crack spacing significantly differ from normal concrete. However, the crack-width-calculation formula in the CECS38-2004 does not consider the influence of the force characteristics of RPC on the key parameters in the crack-width calculation. The stiffening effect of the fibers is taken into account by multiplying a discount factor.

Li [33] calculated the crack width of steel–RPC composite structures according to the UHPFRC-2016, and the results showed that when the cover thickness was small, the calculated crack width obtained according to UHPFRC-2016 significantly differed from the experimentally measured values. The reason may be that the shrinkage strain of RPC reaches more than 700με after high-temperature steam curing, while the longitudinal reinforcement and steel plates limit the free shrinkage of RPC, which results in a certain amount of stress in the steel reinforcement before bearing the external load. The steel-reinforcement stress is a critical parameter in the crack-width calculation, resulting in significant differences between the calculated and measured crack widths. Therefore, for densely reinforced steel–RPC composite plates with small cover thickness, the calculation of reinforcement stresses becomes critical for the calculation of crack width.

The crack-width-calculation formulas in UHPFRC-2016 consider the properties of UHPC materials and the effect of the bridging action of steel fibers on key parameters such as reinforcement stress and average crack spacing; therefore, the code UHPFRC-2016 is subsequently used to further discuss the crack-width-calculation method of the densely reinforced steel–RPC composite structure.

### 4.2. Calculation Method of Reinforcement Stress in Densely Reinforced Steel–RPC Composite Plates

**Figure 17:**
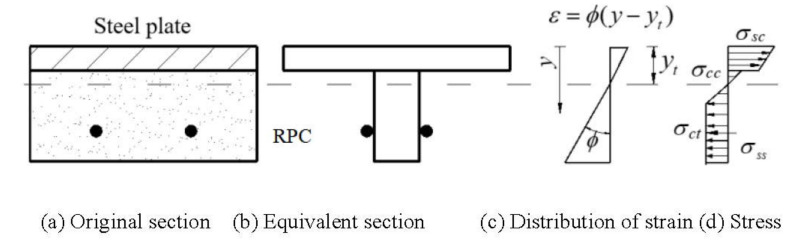
Calculation diagram of reinforcement stress.

According to the test results, when the maximum crack width did not exceed 0.2 mm, the slip between the steel and the RPC layer was relatively small and RPC did not cease to function because of the bridging effect of the steel fibers. Besides, zhang et al. [34] studied the axial tensile properties of RPC with different fiber volume contents, and the results showed that the linear deviating initial crack stress, the visible initial crack stress (crack width reaches to 0.05 mm) and the ultimate peak stress were basically equal when the steel-fiber content was high. Therefore, to simplify the calculation, the following assumptions are made based on the test results and the force characteristics of the steel–RPC composite plate: (1) The strain distribution in the section satisfies the plane-section assumption; (2) The RPC in the tensile area is considered to participate in force, and the stress after the RPC exceeds the proportional limit strain is maintained as the tensile strength. Figure 17 shows a schematic diagram of the reinforcement-stress calculation.

Assuming that the neutral axis lies within the RPC layer, when the section cracks, the equations for the equilibrium of internal forces and bending moments in the section under external loading will be expressed as follows:

(5) $$N_{rt}+N_{ct}+N_{cc}+N_{sc}=0$$

(6) $$M_{rt}+M_{ct}+M_{cc}+M_{sc}-M_{load}=0$$

where $N_{sc}$ and $M_{sc}$ are the internal forces and bending moments borne by the steel plate in the compression area, respectively; $N_{cc}$ and $M_{cc}$ are the internal forces and bending moments borne by the RPC in the compression area, respectively; $N_{ct}$ and $M_{ct}$ are the internal forces and bending moments borne by the RPC in the tension area, respectively; $N_{rt}$ and $M_{rt}$ are the internal forces and bending moments borne by the reinforcement area, respectively; and $M_{load}$ is the bending moment of the section due to the external load.

Figure 17 shows the stress distribution in the section, and the internal forces in Equations (5) and (6) can be obtained:

(7) $$N_{sc}=bE_{s}\Phi h_{s}\left(h_{s}/2-y_{t}\right)$$

(8) $$N_{cc}=-bE_{c}\Phi {\left(h_{s}-y_{t}\right)}^{2}/2$$

(9) $$N_{rt}=A_{s}E_{s}\Phi \left(h_{s}+h_{c}-y_{c}-y_{t}\right)$$

(10) $$M_{sc}=bE_{s}\Phi [y_{t}{}^{3}-{\left(y_{t}-h_{s}\right)}^{3}]/3$$

(11) $$M_{cc}=bE_{c}\Phi {\left(y_{t}-h_{s}\right)}^{3}/3$$

(12) $$M_{rt}=A_{s}E_{s}\Phi {\left(h_{s}+h_{c}-y_{c}-y_{t}\right)}^{2}$$

where: $y_{c}$ is the distance from the shape center of the reinforcement to the RPC surface; b is the width of the combined member; $h_{s}$ is the thickness of the steel plate; $h_{c}$ is the thickness of the RPC layer; $A_{s}$ is the cross-sectional area of the longitudinal reinforcement; $E_{s}$ is the modulus of elasticity of the steel, taken as 206 GPa; $E_{c}$ is the modulus of elasticity of the RPC; ∅ is the bending curvature of the cross-section; $y_{t}$ is the distance from the neutral axis to the bottom surface of the steel plate.

Besides, the internal forces $N_{ct}$ and $M_{ct}$ can be expressed as:

(13) $$N_{ct}=bE_{c}\Phi h_{yt}{}^{2}/2+bf_{ct}\left(h_{s}+h_{c}-h_{yt}-y_{t}\right)$$

(14) $$M_{ct}=bE_{c}\Phi h_{yt}{}^{3}/3+bf_{ct}[{\left(h_{s}+h_{c}-y_{t}\right)}^{2}-h_{yt}{}^{2}]/2$$

where $f_{ct}$ is the axial tensile strength of the RPC, $h_{yt}$ is the height at which the strain of RPC reaches the ultimate axial tensile strain, $h_{yt}=\varepsilon_{ct}/\emptyset$.

**Figure 18:**
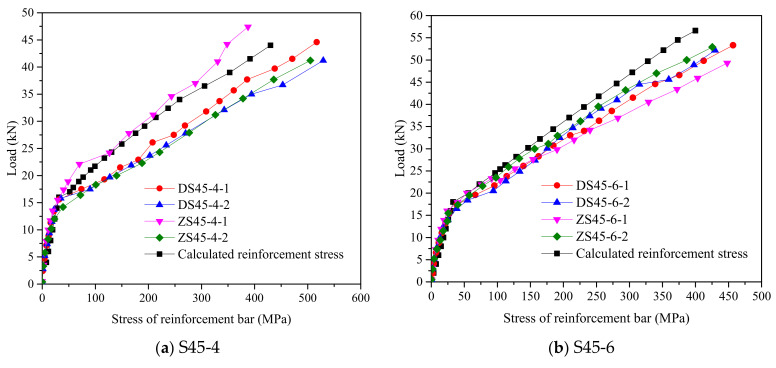
Load–Steel-Stress Curve of members.

Thus, the reinforcement stress can be obtained from Formulas (5)–(14). Figure 18 shows the measured reinforcement stresses and the results of the calculated reinforcement stresses. It can be seen that before the RPC layer cracked, the reinforcement stress increased slowly with the load, and the load–reinforcement-stress curves for the members with the same reinforcement ratio were straight lines and almost coincided. When cracks appeared in the RPC layer, the reinforcement stress rapidly increased, and the load–reinforcement-stress curves were still approximately straight lines. Meanwhile, the differences in the distribution of steel fibers in the RPC and the uncertainty in the location and extension of the cracks had an effect on the reinforcement stresses, resulting in variations between the members. Overall, the calculated and measured reinforcement stresses were in good agreement.

### 4.3. Crack-Width-Calculation Method of Steel–RPC Composite Structures

The effects of RPC performance and steel-fiber action are considered in the UHPFRC-2016 specification. The crack width is calculated as:

(15) $$w_{s}=s_{r,\max,f}\left(\varepsilon_{sm,f}-\varepsilon_{cm,f}\right)$$

(16) $$s_{r,\max,f}=2.55\left(l_{0}+l_{t}\right)$$

(17) $$l_{0}=1.33c/\delta$$

(18) $$\delta =1+0.4\cdot \left(\frac{f_{ctfm}}{K_{global}^{\prime }\cdot f_{ctm,el}}\right)\leq 1.5$$

(19) $$l_{t}=2\cdot \left[0.3\cdot k_{2}\left(1-\frac{f_{ctfm}}{f_{ctm,el}\cdot K_{global}}\right)\frac{1}{\delta \cdot \eta }\right]\frac{\Phi }{\rho_{eff}}\geq \frac{l_{f}}{2}$$

(20) $$\varepsilon_{sm,f}-\varepsilon_{cm,f}=\frac{\sigma_{s}}{E_{s}}-\frac{f_{ctfm}}{E_{cm}\cdot K_{global}}-\frac{1}{E_{s}}\left[k_{t}\left(f_{ctm,el}-\frac{f_{ctfm}}{k}\right)\cdot \left(\frac{1}{\rho_{eff}}+\frac{E_{s}}{E_{cm}}\right)\right]$$

(21) $$w_{t}=w_{s}\cdot \left(h-x-x^{\prime }\right)/\left(d-x-x^{\prime }\right)$$

where $w_{s}$ represents the maximum crack width at the level of longitudinal reinforcement, $w_{t}$ represents the maximum crack width distributed on the top surface of RPC under highest tension; $S_{r,\max,f}$ is the maximum crack spacing; $\varepsilon_{sm,f}$ is the average strain of reinforcement between cracks; $\varepsilon_{cm,f}$ is the average strain of RPC material between cracks; ∅ is the diameter of the reinforcement; η is the bonding factor, taken as 2.25; c is the net protective-layer thickness; δ is the enhancement factor of the fiber to the thickness and bond strength of the protective layer; $l_{t}$ is the transfer length; $f_{ctm,el}$ is the average of the RPC tensile elastic ultimate strength. $f_{ctfm}$ is the average value of the strength after cracking, which is calculated as 75% of $f_{ctm,el}$; $\rho_{eff}=A_{s}/A_{c,eff}$ is the effective reinforcement ratio in the tension zone; $\sigma_{s}$ is the stress of the crack-section reinforcement; $K_{global}$ is the longitudinal reinforcement orientation factor, which is 1.75 for local loads and 1.25 for other loads; $K_{global}^{\prime }$ is the orientation coefficient of the transverse steel bars; $k_{t}$ is the load acting-time coefficient. For short-term loads, $k_{t}=0.6$; for long-term loads or high-amplitude repeated loads, $k_{t}=0.4$; h is the total height of the cross section; d is the efficient depth of the cross section; x is the compressed height; $x^{\prime }$ is the uncracked height under tension.

As the influence of fiber type is not directly considered in the crack-width calculation formulas in UHPFRC-2016, this paper takes the material performance results of the EHHF members for calculation. The reinforcement stress is calculated by Equations (5)–(14) and other parameters in Formulas (15)–(21) are taken according to the related specification. The calculated crack-width curves are shown in Figure 16. The calculated results and the measured crack-width curves are in good agreement. Therefore, the proposed crack-width-calculation method of densely reinforced steel–RPC composite structure can provide guidance for engineering design.

## 5. Conclusions

A series of material-property tests and transverse-bending tests of steel–RPC composite structure with different fiber parameters is conducted in this paper. In addition, the modified crack-width-calculation method of densely reinforced composite structure is proposed. The main conclusions are as follows:

(1) Increasing the fiber content can significantly increase the compressive strength, bending initial crack strength, flexural strength and the direct-tensile stress at each characteristic point of specimens. Meanwhile, the hybrid fiber was more effective than the long straight type of fiber. The specimen with a fiber volume content of 2% had a significantly lower modulus of elasticity than the other members. Strain hardening can occur in RPC materials when the steel-fiber volume content is 2%.
(2) The cracking stresses of EHHF members were 6.7–26.5% higher than in the long-straight hybrid-fiber members, while the ultimate load capacity was essentially the same. When the longitudinal reinforcement ratio increased from 3.5% to 5.2%, the cracking stress and ultimate bearing capacity of the members increased by 12.4–33.3%, 25.5–29.2%, respectively.
(3) The steel–RPC composite plates failed due to the yield of longitudinal reinforcement bars. After that, the displacement and the crack width rapidly increased, but the bearing capacity of the member remained basically unchanged. For EHHF members, the crack-expansion speed was significantly less than for long-straight hybrid fiber members.
(4) The distribution of cracks in the pure-bending section of the reinforced steel–RPC composite plate was dense and relatively uniform, and the higher the reinforcement ratio, the denser the cracks. When the reinforcement ratio was the same, the straight hybrid-fiber members were more densely cracked than the EHHF members.
(5) Based on the experimental results, the calculation methods of reinforcement stress and crack width of densely reinforced steel–RPC composite structure are proposed. The calculated results of reinforcement stress and maximum crack width of RPC are in good agreement with the actual measured values.

## Figures and Tables

**Table 1:** Design of steel fiber composite parameters.

Specimen Group	Steel Fiber Diameter (mm)	Steel Fiber Length (mm)	Steel Fiber Type	Steel Fiber Volume Content (%)	
long-straight steel fiber	0.2	13	long-straight	2	
	0.2	13	long-straight	2.5	
	0.2	13	long-straight	3	
	0.2	13	long-straight	3.5	
Hybrid steel fiber	0.12	8	long-straight	1	2
	0.2	13	End hook	1	
	0.12	8	long-straight	1	2.5
	0.2	13	End hook	1.5	
	0.12	8	long-straight	1.5	3
	0.2	13	End hook	1.5	
	0.12	8	long-straight	1.5	3.5
	0.2	13	End hook	2	

**Table 2:** The characteristics of the RPC–steel fiber of the tensile test.

Specimen Group	Steel Fiber Type	Fiber Volume Content (%)	Length × Diameter (mm)	Length to Diameter Ratio	Density (kg/m^3^)	Tensile Strength (MPa)	Modulus of Elasticity (GPa)
H-A	End hook	2	13 × 0.2	59	7850	>2000	200
S-A	long-straight	2	13 × 0.2	59			
S-B	long-straight	2	13 × 0.15	87			
M-A	long-straight	0.4	8 × 0.12	67			
	long-straight	1.6	13 × 0.15	87			

Remark: “ H “ for end hooked fibers, “ S “ for long-straight fibers, “ M “ for hybrid fibers, “ A “ and “ B “ for different fiber length to diameter ratios in the designation of the specimen number.

**Table 3:** The test results of material properties.

Steel Fiber Type	Fiber Volume Content (%)	Compressive Strength (MPa)	Modulus of Elasticity (GPa)	Bending Initial Cracking Point		The Bending Limit Point	
				Load (kN)	Strength (MPa)	Load (kN)	Strength (MPa)
long-straight fiber	2	137.1	42.7	37.9	11.4	78.9	23.7
	2.5	140.7	43.9	39.2	11.7	88.0	26.4
	3	141.3	44.8	40.2	12.1	94.6	28.4
	3.5	145.6	44.8	40.0	12.0	100.1	30.0
Hybrid steel fiber	2	139.6	42.5	37.1	11.1	84.0	25.2
	2.5	144.0	45.3	41.1	12.3	93.6	28.0
	3	148.1	45.6	41.0	12.3	102.1	30.6
	3.5	154.7	45.8	44.2	13.3	104.9	31.4

**Table 4:** Parameter of members.

Member Name	Fiber Type	Fiber Volume Content and Size (Length × Diameter)	Number of Reinforcement Bars	Reinforcement Ratio
ZS45-4-1	Straight hybrid fiber	2% straight fibers (13 mm × 0.2 mm) + 1.5% straight fibers (8 mm × 0.12 mm)	4	3.5%
ZS45-4-2				
ZS45-6-1			6	5.2%
ZS45-6-2				
DS45-4-1	End hook hybrid fiber (EHHF)	2% end hook fibers (13 mm × 0.2 mm) + 1.5% straight fibers (8 mm × 0.12 mm)	4	3.5%
DS45-4-2				
DS45-6-1			6	5.2%
DS45-6-2				

Sample identification: ZS/DS in the table represents linear hybrid-fiber members and EHHF members, respectively, 45 mm represents the thickness of the RPC layer, 4/6 represents the number of longitudinal reinforcement bars arranged in RPC layer, and 1/2 represents the number of the member.

**Table 5:** Main test results.

Name of Member	Cracking Load (kN)	Ultimate Load (kN)	Cracking Stress (MPa)	Average Cracking Stress (MPa)	Average Ultimate Load (kN)
ZS45-4-1	25	57.3	22.4	22.5	58.9
ZS45-4-2	25.1	60.5	22.5		
DS45-4-1	26.9	60.4	24.1	24	59.5
DS45-4-2	26.5	58.7	23.9		
ZS45-6-1	30.6	75.5	26.1	25.3	76.1
ZS45-6-2	28.6	76.8	24.4		
DS45-6-1	36.9	74.5	31.5	32	74.7
DS45-6-2	38.1	74.9	32.5		

**Table 6:** Average crack spacing and number of main cracks.

Name of Member	Average Crack Spacing (mm)				Number of Main Cracks
	$w_{max}\;=\;0.05$	$w_{max}\;=\;0.1$	$w_{max}\;=\;0.2$	Ultimate Load Bearing Condition	
ZS45-4-1	66.7	50	36.4	36.4	3
ZS45-4-2	80	57.1	40	36.4	3
DS45-4-1	80	66.7	40	40	2
DS45-4-2	66.7	57.1	44.4	40	3
ZS45-6-1	44.4	40	30.8	30.8	2
ZS45-6-2	50	44.4	33.3	33.3	2
DS45-6-1	50	40	33.3	33.3	2
DS45-6-2	57.1	50	40	36.4	3

## Data Availability

Not applicable.
